# Supplementary material for: Brain regions modulated during covert visual attention in the macaque
Source: Sci Rep. 2018 Oct 15;8:15237. doi: 10.1038/s41598-018-33567-9 (PMC6189039; doi:10.1038/s41598-018-33567-9)
Supplement: Supplementary file 1 — Supplementary Information [file 41598_2018_33567_MOESM1_ESM.pdf]

Brain regions modulated during covert visual attention in the macaque

Amarender R. Bogadhi, Anil Bollimunta, David A. Leopold, Richard J. Krauzlis

Supplementary figures

Figure S1. Activations during Stimulus mapping experiment (related to Fig. 2)

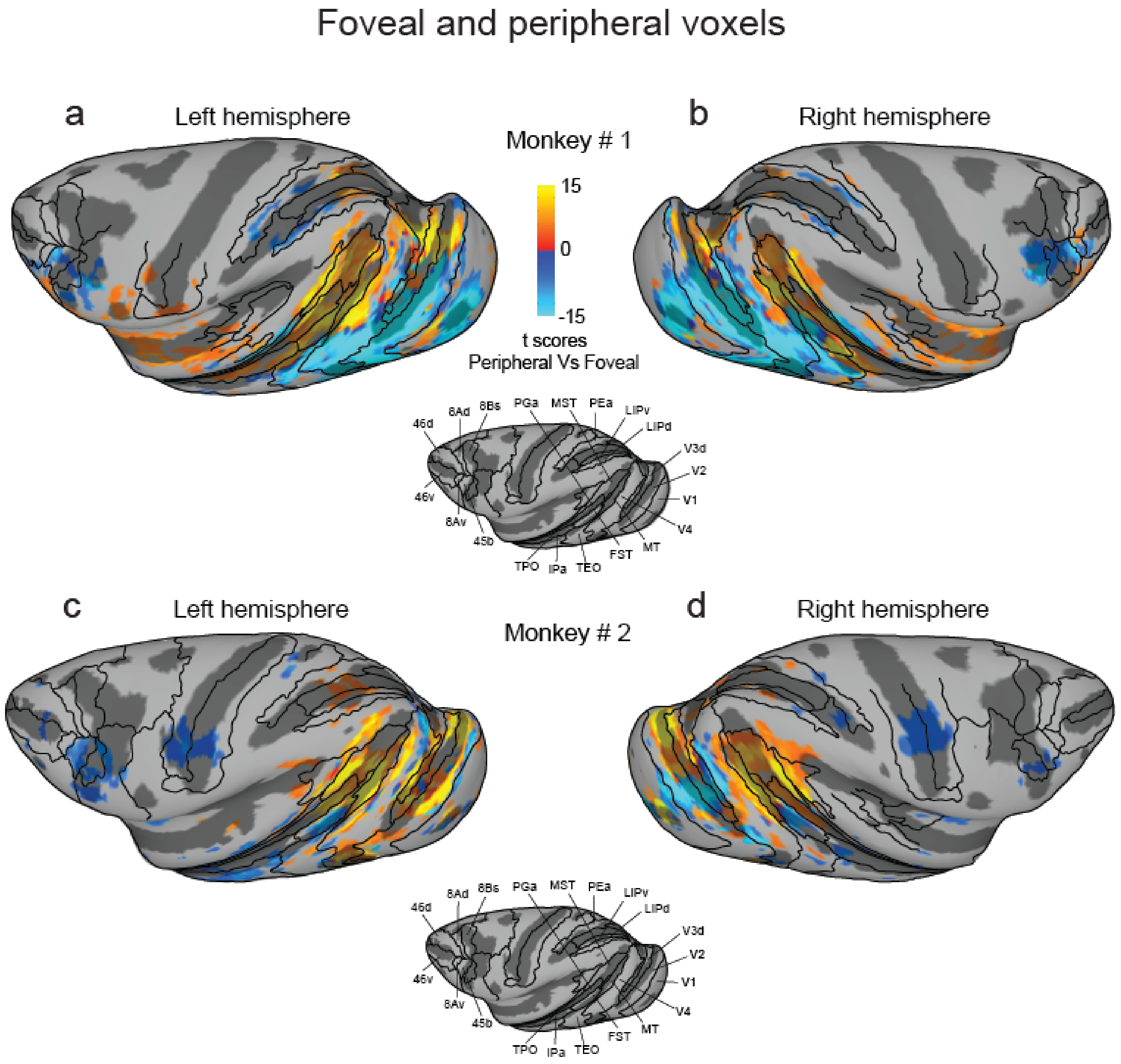

Figure S1. (a – d) T-scores contrasting Peripheral and Central stimulus conditions during stimulus mapping experiment show activations in Peripheral voxels (red to yellow) and Central 2^0 voxels (blue to cyan) in left (a, c) and right (b, d) hemispheres of monkey # 1 and monkey # 2 respectively. Anatomical boundaries are labeled for the left hemisphere. T-scores were corrected for multiple comparisons (Bonferroni correction; $p < 0.05$, $|t\text{-score}| > 5.02$). The foveal activations in areas TEO and V4 of monkey #2 were much sparser compared to monkey #1 because of the MR signal dropout at the lateral edges of the implant that overlapped with lateral visual cortical areas representing fovea locations.

Figure S2. Cortical maps of attention-related activation (unthresholded) (related to Fig. 3)

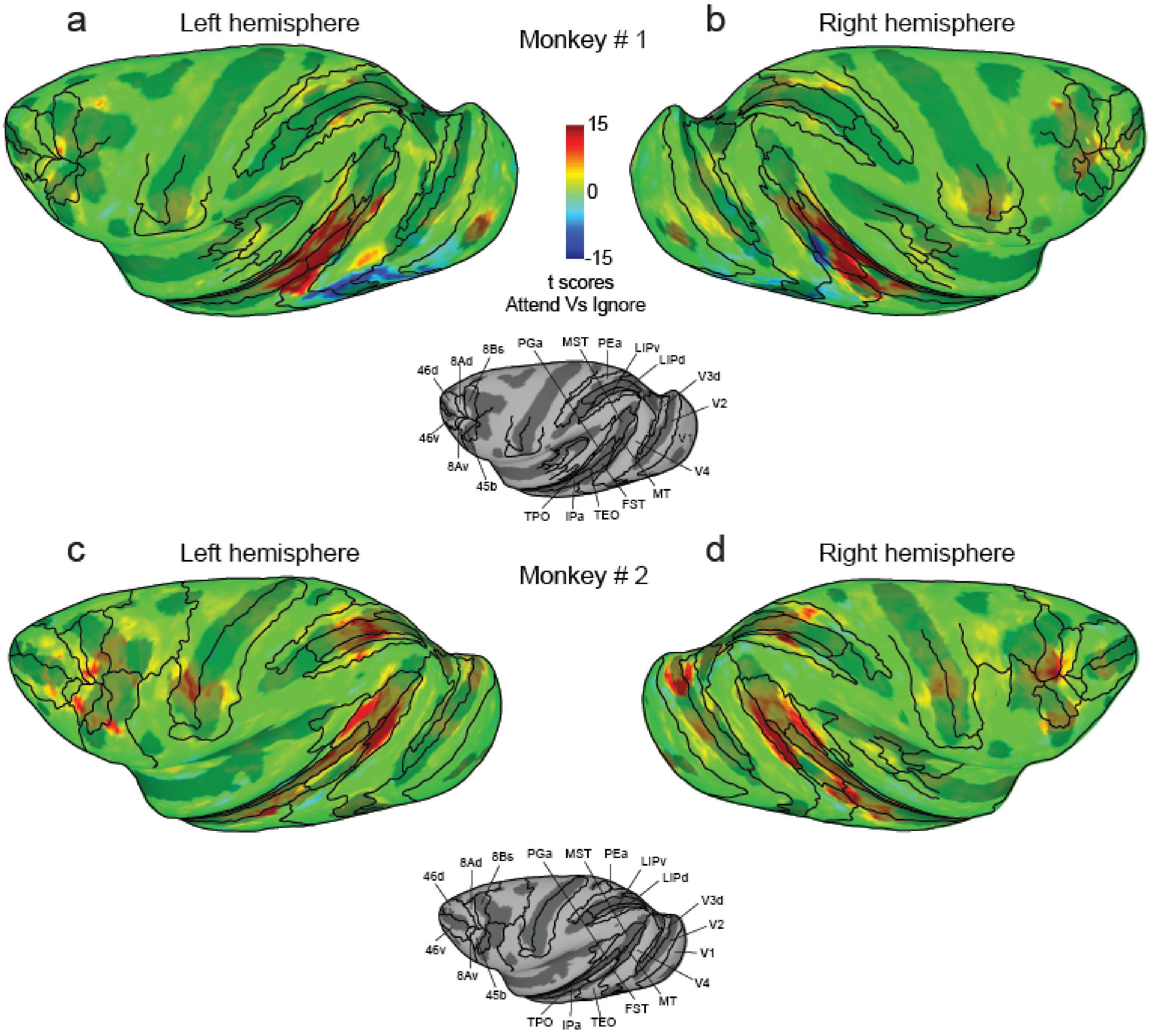

Figure S2. T-scores (unthresholded) contrasting Attend and Ignore tasks were projected onto inflated cortical surfaces of D99 in each monkey’s native space along with anatomical boundaries (black contours). (a, b) Inflated cortical maps of t-scores showing attention-related activation in left (a) and right (b) hemispheres of monkey # 1.

Anatomical boundaries are labeled for the left hemisphere in monkey # 1. (c, d) Inflated cortical maps of t-scores showing attention-related activation in left (c) and right (d) hemispheres of monkey # 2. Anatomical boundaries are labeled for the left hemisphere in monkey # 2.

Figure S3. Activations during Ignore and Attend tasks using second-order orientation stimulus (related to Fig. 8)

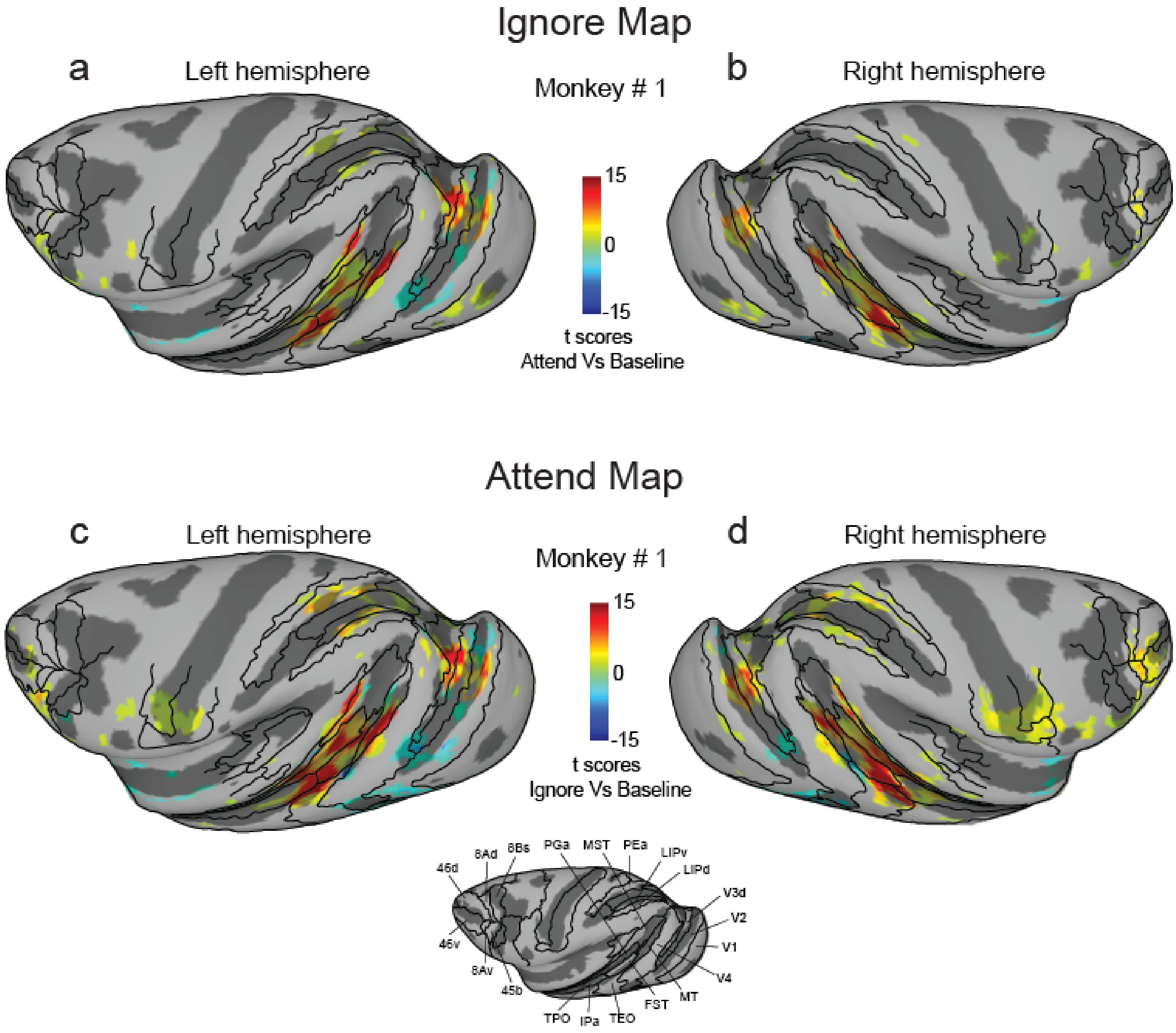

Figure S3. (a, b) T-scores contrasting Ignore and baseline tasks show activations during Ignore task in left (a) and right (b) hemispheres of monkey # 1. (c, d) T-scores contrasting Attend and baseline tasks show activations during Attend task in left (c) and right (d) hemispheres of monkey # 1. Anatomical boundaries are labeled for the left hemisphere. T-scores were corrected for multiple comparisons (Bonferroni correction; p < 0.05, |t-score| > 5.02).

Figure S4. Stability of attention-related modulation to second-order orientation stimulus in the aFST/IPa region (related to Fig. 8)

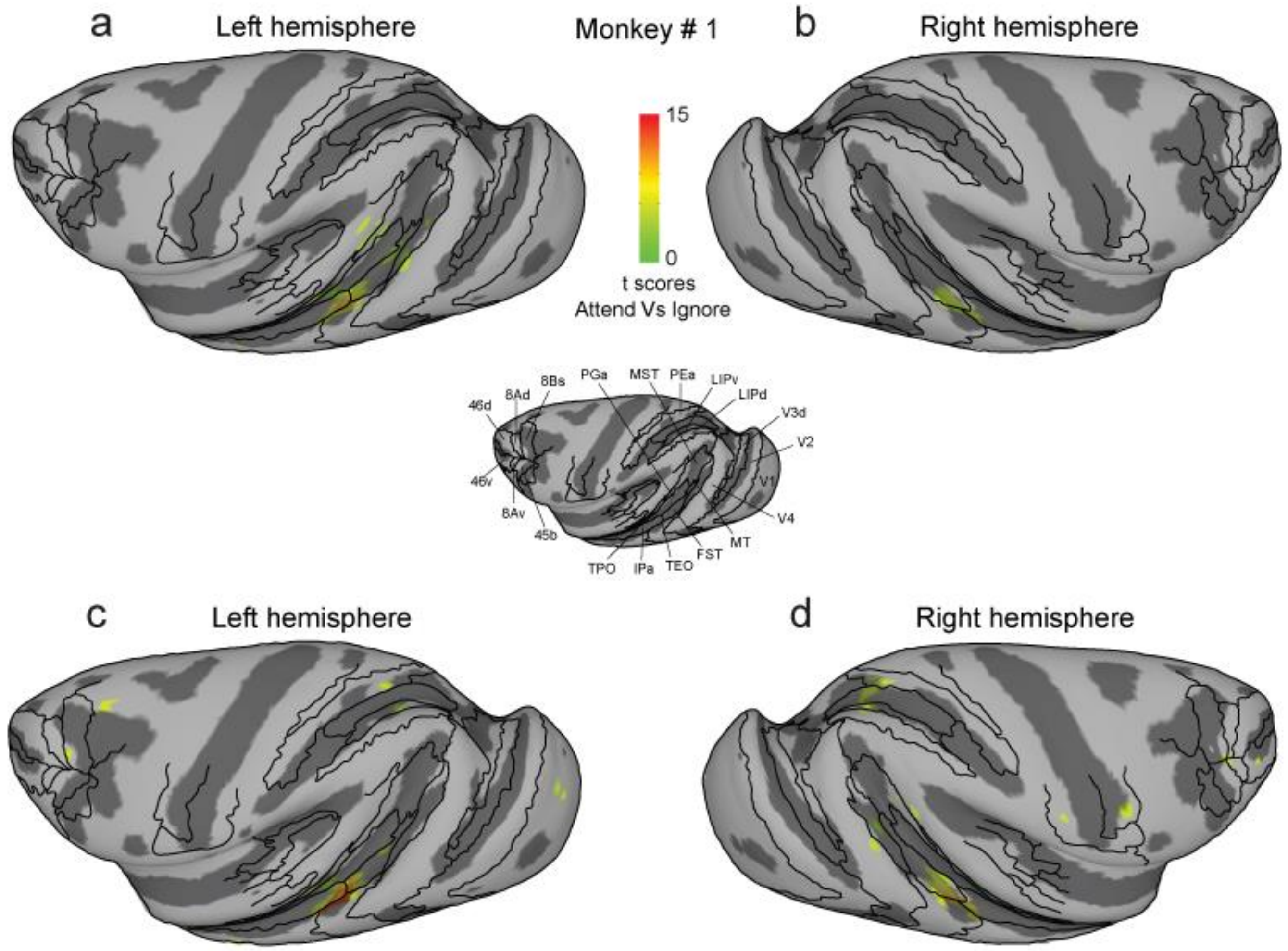

Figure S4. We verified the stability of attention-related modulation to second-order orientation stimulus in the aFST/IPa region by splitting the data shown in figure 8. T-scores contrasting Attend and Ignore tasks described in figure 7 were projected onto inflated cortical surfaces of D99 in native space of monkey # 1 along with anatomical boundaries (black contours). Inflated cortical maps of t-scores (Bonferroni correction; $p < 0.05$, t-score > 5.02) show attention-related modulation in left (a, c) and right (b, d) hemispheres of monkey # 1 for the first half (a, b) and second half (c, d) of the dataset shown in figure 8.

Supplementary methods

Experimental apparatus

Monkeys were seated and head-fixed in a custom-built MR-safe chair with a joystick attached inside the chair. Stimuli were back projected on to a screen placed inside the bore of the vertical magnet using an Epson projector controlled by a Windows 2007 machine running MATLAB R2012b (The Mathworks) with the psychophysics toolbox extensions. The timing of the stimuli and events were controlled by a QNX system running QPCS. Monkey viewed the screen through a mirror placed in front at a 45° angle. The total viewing distance of the screen was 53 cm. Eye movements were acquired and monitored in the scanner using an iView system (Version 2.4, SensoMotoric Instruments). Eye signal was calibrated at the beginning of each session. Joystick presses and releases were detected using a MRI-compatible custom device

that operated by detecting deflections in an optical beam and provided signals about timing but not kinematics of the joystick press and release. Joystick was calibrated once at the beginning of the experiments.

Random dot motion stimuli

The random dot motion stimuli were circular patches of moving dots, with the direction of motion of each dot drawn from a normal distribution with a mean value (defined as the patch motion direction) at 30° above horizontal and a 16° standard deviation. The lifetime (10 frames, 100 ms), density (25 dots/^{o2}/s), and speed of the dots (15 ^{o}/s) were held constant. The radius of the aperture was set to 3°. Luminance of each moving dot in the motion patches was 50 cd/m^2. The change in direction of motion (Δ) was 1 ± 0.25 standard deviations for both monkeys across sessions.

Fixation spot stimulus

The size of the fixation spot was 0.23^o and the size of the central cue was 0.35^o. The background luminance of the screen was 14 cd/m^2 and the luminance of the fixation spot was 50 cd/m^2. The luminance change in fixation spot during Baseline and FA trials was 1-2 cd/m^2 across sessions for both monkeys.

Stimulus mapping experiment

To identify voxels responding to foveal and peripheral stimuli locations, a flickering checker board stimulus (4 Hz) that has concentric rings of 2° width spanning up to 12° eccentricity was used. In foveal visual stimulation blocks, the checker board

stimulus was masked everywhere except for the central 2º radius. In peripheral visual stimulation blocks, the checker board stimulus was masked everywhere except for the two eccentric stimulus locations used in the attention tasks. The foveal and peripheral stimulation blocks (20 s duration) were interleaved with fixation blocks (10 s duration). A total of 258 runs (150 in Monkey #1; 108 in Monkey # 2) were collected in both monkeys across 13 sessions. Functional maps showing peripheral and foveal voxels were created using the same methods as described for creating functional maps for the attention tasks (Fig. S2).
